# Buffalo General Hospital—Inspection of the Military Wards and Sick and Wounded Soldiers

**Published:** 1864-09

**Authors:** 


*Buffalo General Hospital—Inspection of the Military Wards and Sick and Wounded Soldiers.*—A commission of inspection, consisting of Gen. Fitz Hugh Warren of Iowa, well known to many of our citizens, Surgeon Craven, Medical Director of the 10th corps, and Capt. ———, are on a visit to the United States Hospitals in this vicinity, for the purpose of ascertaining their condition, and with view of sending to the front any who may be found sufficiently recovered to be able to do active military duty. The Buffalo General Hospital was inspected this morning, (Sept. 16,) and was complimented in the highest terms—as unsurpassed for order, neatness, ventillation, and all the attractions of a well arranged, well managed home for sick and wounded soldiers. The medical wards under the care of Dr. S. F. Mixer, and the surgical wards under the care of Dr. Sandford Eastman, were pronounced in condition to reflect the highest credit upon their fidelity and capacity, and the members of the commission were forward in expressing their hearty approval. Dr. Brown, the House Physician, and Mr. Brayley, the Superintendent, received their full share of compliment. All friends of the Hospital and all interested in the care of soldiers will be much gratified by the report.

The wards are now full, and it is becoming a matter of pressing importance that more room be provided. The building is too crowded for perfect hygienic condition, and immediate provision should be made for accommotion of more soldiers and general patients. Applicants for private rooms are constantly disappointed, and general patients for the wards can hardly be accommodated. The front building to the Buffalo General Hospital should be immediately commenced. The attention of the Trustees is earnestly called to it.

